# Madelung Deformity of the Wrist Managed Conservatively

**DOI:** 10.7759/cureus.8225

**Published:** 2020-05-21

**Authors:** Pratyush Shahi, Aarushi Sudan, Apoorv Sehgal, Debasish Meher, Umesh Meena

**Affiliations:** 1 Orthopaedics, University College of Medical Sciences and Guru Teg Bahadur Hospital, Delhi, IND; 2 Psychiatry, University College of Medical Sciences and Guru Teg Bahadur Hospital, Delhi, IND

**Keywords:** madelung deformity, wrist, volar-ulnar distal radial physis, adolescence, conservative management, vickers ligament, dorsal subluxation of distal ulna

## Abstract

A 19-year-old female presented with pain, deformity, and slightly restricted left wrist motion for five years with gradual progression. Physical examination revealed volar subluxation of the left hand, dorsally prominent ulnar styloid, radial and dorsal bowing of the distal forearm, and mild restriction in wrist dorsiflexion. Radiographs showed a failure of ossification of the ulnar side of the distal radial epiphysis, increased radial inclination angle, dorsal subluxation of the distal ulna, V-shaped proximal carpal row due to proximal migration of the lunate, and increased interosseous space. A diagnosis of Madelung deformity of the left wrist was made. Conservative management with oral analgesics, activity restriction, and a volar splint was done as the patient was skeletally mature, had only mild pain with no functional limitation or gross deformity. At the six-month follow-up, she was doing well with decreased pain and no new complaints.

## Introduction

Madelung deformity (MD) is a rare entity caused by premature growth arrest of the volar-ulnar distal radial physis. Patients generally present in adolescence with pain, deformity, and restricted range of motion of the wrist joint [1]. We, through this case report, aim to highlight the presentation, examination and radiographic findings, and treatment modalities of this rare deformity.

## Case presentation

**Figure 1:**
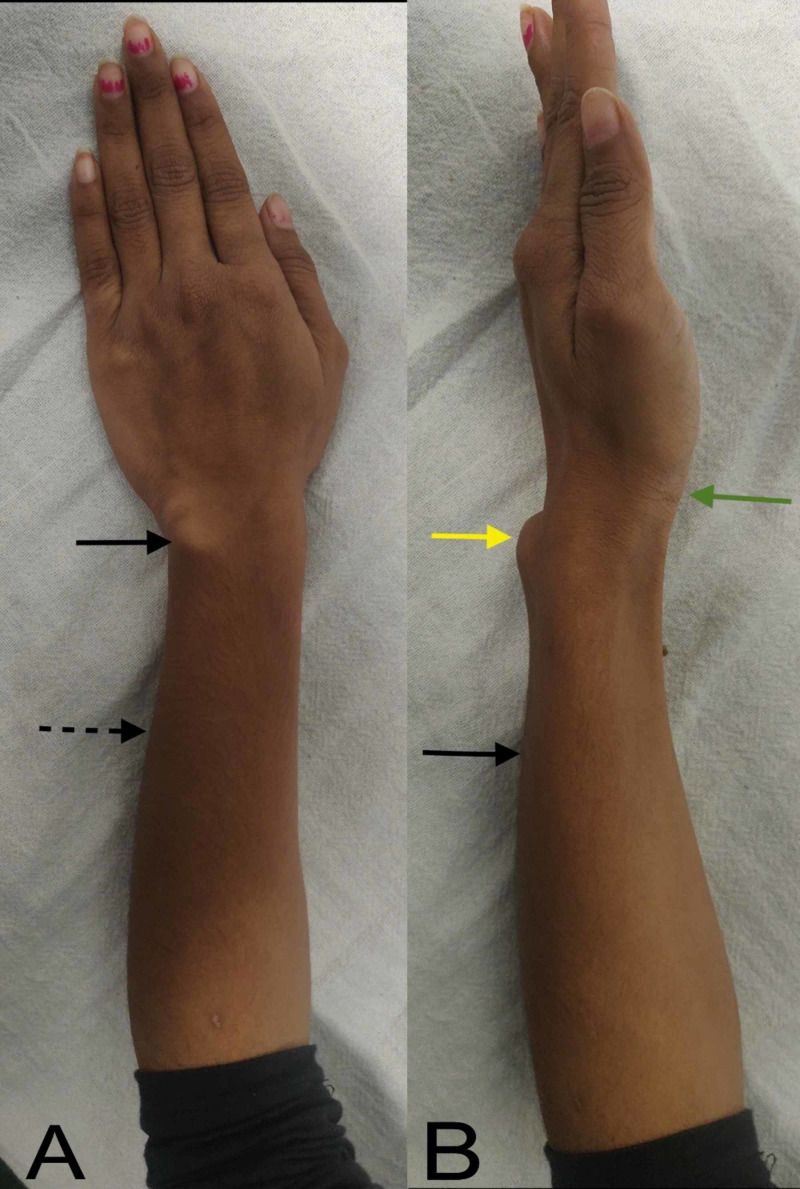
Clinical assessment. (A) Ulnar bowing of the forearm (dashed arrow) and prominent ulnar styloid (solid arrow). (B) Dorsal bowing of the forearm (black arrow), dorsally subluxated ulnar styloid (yellow arrow), and volarly subluxated hand (green arrow).

A 19-year-old female presented to us with pain, deformity, and slight limitation of motion of the left wrist. The symptoms had started about five years back and had gradually progressed since. Pain used to increase with strenuous activity. There was no involvement of any other joint. Physical examination revealed palmar subluxation of the left hand, dorsal prominence of the ulnar styloid, and ulnar and dorsal bowing of the forearm (Figure 1).

**Figure 2:**
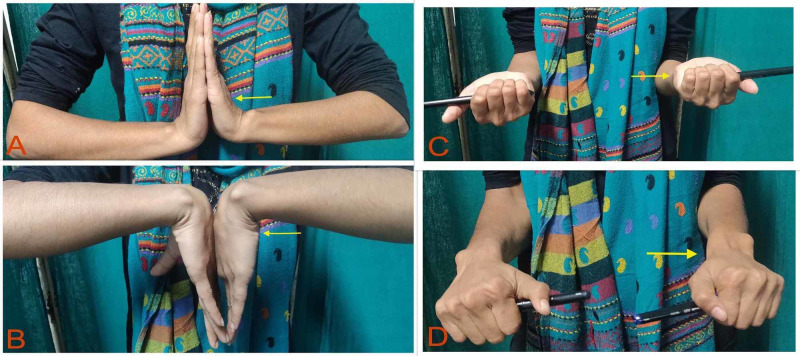
Range of motion of the wrist joint. (A) Slightly restricted dorsiflexion at the left wrist. (B-D) Images show comparable palmar flexion, supination, and pronation, respectively.

Dorsiflexion of the left wrist was slightly restricted, whereas palmar flexion, pronation, and supination were comparable to the right side (Figure 2). 

**Figure 3:**
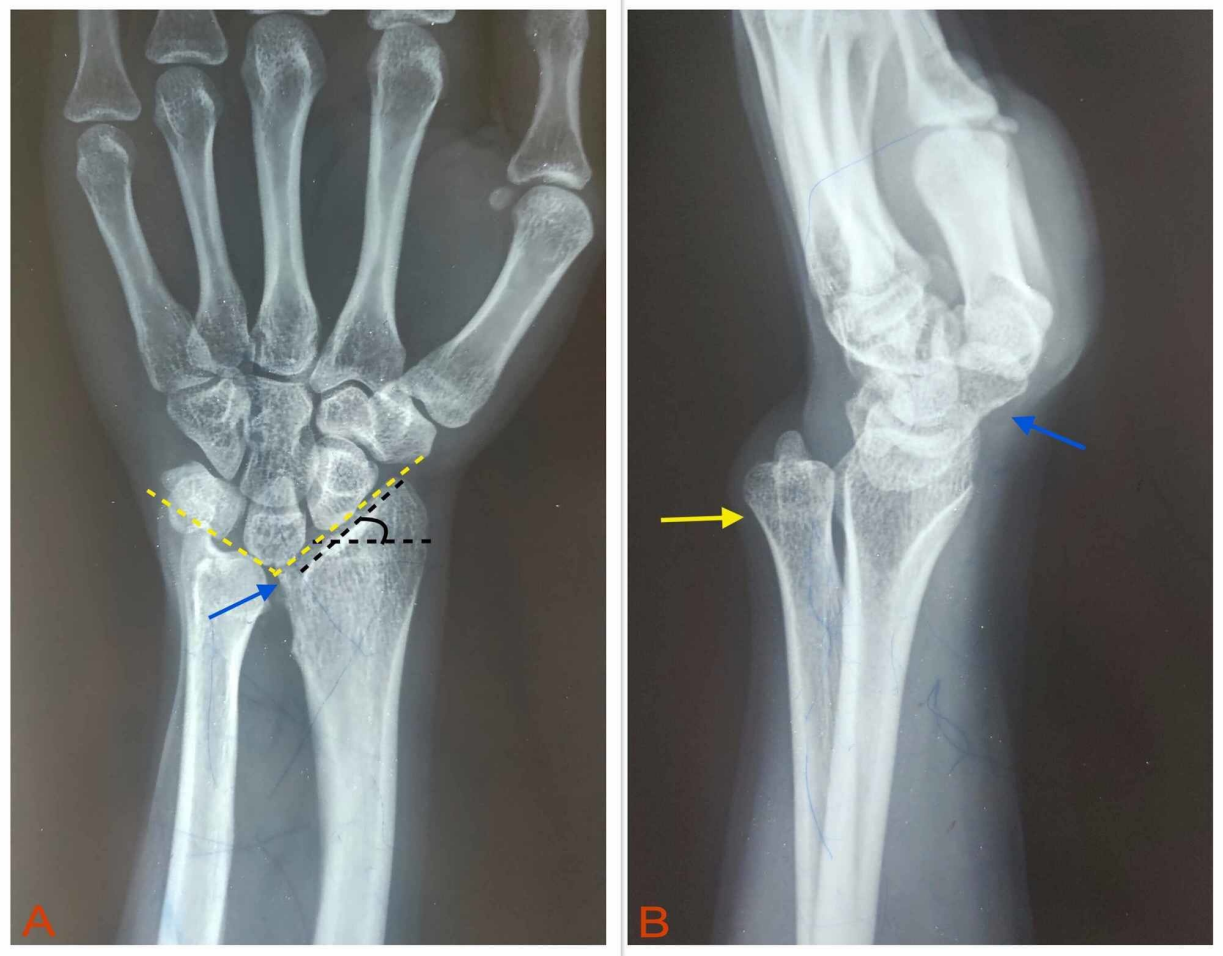
Radiographic assessment. (A) Anteroposterior view shows a failure of ossification of the ulnar side of the distal radial epiphysis (blue arrow), increased radial inclination (angle formed by the black dashed lines), and proximal migration of the lunate causing V-shaped carpus (yellow dashed lines). (B) Lateral view shows dorsally subluxated distal ulna (yellow arrow) and volarly subluxated hand (blue arrow).

Anteroposterior and lateral view X-rays of the left wrist showed a failure of ossification of the ulnar side of the distal radial epiphysis, increased radial inclination, dorsal subluxation of the distal ulna, V-shaped proximal carpal row, and increased interosseous space (Figure 3). A diagnosis of MD of the left wrist was made. 

The patient was conservatively managed after discussing the various treatment modalities with her and the parents. She was prescribed oral analgesics, activity restriction, and a volar splint. At the six-month follow-up, her pain had reduced and she had no complaints.

## Discussion

First described by Otto Madelung in 1878, MD of the wrist results from premature closure of the volar-ulnar distal radial physis [2]. It is a rare entity with a prevalence of less than 2%. There is a female preponderance with a female to male ratio of 3-5:1. The deformity is generally bilateral (our case had unilateral deformity) [3]. 

Henry and Thorburn described four etiologic factors of MD: posttraumatic, dysplastic, genetic, and primary [4]. Posttraumatic can be due to repetitive trauma (as seen in gymnasts) or a single event. The most common dysplasia associated with MD is Leri-Weill dyschondrosteosis, which is characterized by mesomelic short stature due to a mutation in the SHOX gene [5]. Other bone dysplasias associated with MD are multiple hereditary osteochondromatosis, Ollier's disease, achondroplasia, multiple epiphyseal dysplasias, and mucopolysaccharidoses. MD is also frequently seen in Turner’s syndrome. Vickers ligament, considered as a short volar radiolunate ligament, can compress the volar-ulnar physis of the distal radius and hence is thought to be a causative factor of MD [6].

Remaining asymptomatic in childhood, patients typically present in adolescence with the pubertal growth spurt leading to an increase in the deformity, and associated pain and restriction of movement. As a result of premature growth arrest of the volar-ulnar distal radial physis, there is an increased volar tilt and inclination of the distal radial articular surface, proximal migration of the lunate causing a triangulation of the carpus, and dorsal subluxation of the distal ulna [7].

Treatment is either surgical or non-surgical. Non-surgical treatment includes non-steroidal anti-inflammatory drugs (NSAIDs), activity modification, and a resting splint. Surgical treatment is indicated for pain, instability, and restricted wrist movements after a trial of conservative treatment and includes Vickers physiolysis (excision of the Vickers ligament and the abnormal volar-ulnar distal radial physis), radial corrective osteotomy (to correct the position of the articular surface), and distal ulnar shortening osteotomy (in cases with positive ulnar variance) [8]. Conservative management was done in our case as the patient was skeletally mature, had only mild pain with no functional limitation or gross deformity. 

## Conclusions

MD of the wrist results from premature closure of the volar-ulnar distal radial physis and is characterized by increased volar tilt and inclination of the distal radial articular surface, triangulation of the carpus, and dorsal subluxation of the distal ulna. Patients generally present in adolescence with pain, deformity, and restricted wrist movements. Treatment can be surgical or non-surgical.
